# A Single-Surgeon Experience Transitioning to Total Arterial Revascularization

**DOI:** 10.3390/jcm13164831

**Published:** 2024-08-16

**Authors:** Dwight D. Harris, Louis Chu, Sharif A. Sabe, Michelle Doherty, Venkatachalam Senthilnathan

**Affiliations:** Division of Cardiac Surgery, Beth Israel Deaconess Medical Center, Harvard Medical School, Boston, MA 02215, USA; lchu@bidmc.harvard.edu (L.C.); ssabe@bidmc.harvard.edu (S.A.S.); mdohert1@bidmc.harvard.edu (M.D.); vsenthil@bidmc.harvard.edu (V.S.)

**Keywords:** Total Arterial Revascularization, CABG, coronary artery disease, internal mammary artery

## Abstract

**Background:** Coronary artery bypass grafting remains the standard of care for advanced and multifocal coronary artery disease; however, for patients that are surgical candidates, total arterial revascularization (TAR) remains underutilized due to concerns such as sternal wound infections and the learning curve. We present the results of a large cohort of mid-career surgeons transitioning to TAR, focusing on short-term outcomes and the learning curve. **Methods:** The surgeons transitioned to using TAR as the preferred revascularization technique in August of 2017. The Society of Thoracic Surgeons database was reviewed to identify all patients who underwent isolated non-emergent CABG performed by a single surgeon from January 2014 through January 2022. Patients were divided into two groups—those who had TAR and those who had traditional CABG using one internal mammary artery and vein grafts (IMA-SVG). **Results:** Eight hundred ninety-eight patients meet inclusion criteria (458 IMA-SVG and 440 TAR). The TAR group had slightly longer cardiopulmonary bypass time, cross clamp times, and operative times (all *p* < 0.05); however, ICU stay was shorter and 30-day readmission rate was lower for TAR compared to IMA-SVG (all *p* < 0.05). The TAR group also required fewer postoperative transfusions (*p* = 0.005). There was no difference in prolonged intubation, stroke, length of stay, mortality, or sternal wound complications between groups (all *p* > 0.05). The average TAR was 30 min longer; however, learning curves, stratified by number of grafts placed, showed no significant learning curve associated with TAR. **Conclusions:** An experienced surgeon transitioning from IMA-SVG to TAR slightly increases operative time, but decreases ICU stay, readmissions, and postoperative transfusions with no significant difference in rates of immediate post-operative complications or 30-day mortality, with a minimal learning curve.

## 1. Introduction

Coronary artery disease (CAD) remains the leading cause of mortality worldwide. The management of CAD is a significant financial and resource burden on the health care system [1,2]. The American Heart Association and the Centers for Disease Control indicate that over 20 million Americans are afflicted with CAD and that a myocardial infarction occurs every 40 s in the United States alone [3,4]. Internationally, cardiovascular disease has a similar prevalence, with over 9 million deaths attributed to CAD in 2021 alone, making it the leading cause of death worldwide [5,6]. There have been many advancements in the treatment of advanced CAD with medical management; however, for patients that are candidates, surgical or percutaneous revascularization remains the standard of care [7,8,9,10,11,12]. Coronary artery bypass grafting (CABG) remains an important part of the treatment algorithm for advanced and multifocal CAD with over 300,000 CABGs performed each year in the United States [8]. Traditional CABG utilizes one internal mammary artery and saphenous vein graft (IMA-SVG) [13]. It has long been known that the left internal mammary artery has superior patency, likely due to the arterial vessel wall and the decreased rates of maladaptive intimal hyperplasia as compared to vein grafts [14,15]. Due to concern about the longevity of vein grafts, there is growing interest in utilizing multiple arterial grafts and total arterial revascularization (TAR) [16]. TAR utilizes any combination of mammary and radial arteries to avoid using vein grafts.

TAR has been associated with improved long-term freedom from major cardiac events, including myocardial infarction, cerebrovascular events, and death [17]. This includes a metanalysis of 23 retrospective studies showing improved longer survival with TAR [18]. However, despite multiple retrospective studies and two randomized controlled trials demonstrating the long-term benefits of TAR, TAR remains underutilized [19,20,21,22,23,24,25]. In the Unites States, over 300,000 CABGs are performed each year, but only a small fraction of them are performed using multiple arterial grafts, with either bilateral internal mammary arteries (BIMAs) or radial arteries [26]. This would suggest that an even smaller percentage of surgeons are utilizing TAR. The underutilization of TAR is due to concerns such as sternal wound infections, patient selection, and the learning curve [27,28]. 

The current literature on TAR incudes single center, observational, and randomized controlled trials [19,20,21,22,23,24,29]. However, it is important to consider the experience of individual cardiac surgeons transitioning to TAR. We believe the literature is lacking a large single-surgeon experience particularly focused on the learning curve. We believe that by looking at the single-surgeon experience, we remove the variability related to technical skill and patient-specific care that could be seen in other studies. While there have been large studies focused on outcomes, we present the largest reported experience of single mid-career surgeons transitioning to TAR, focusing on short-term outcomes and the learning curve.

## 2. Methods

### 2.1. Study Design and Data Collection

The Society of Thoracic Surgeons database (STS) was reviewed to identify all patients over 18 years of age who underwent isolated CABG (without other cardiac surgical procedures) performed by a single surgeon at a large academic medical center from January 2014 through January 2022. Patients were divided into two groups—those who had traditional CABG using one internal mammary artery and any number of vein grafts and those who had TAR with any combination of mammary and radial arteries. Chart review was performed using our electronic medical records, and data collected included patient demographics, comorbidities (hypertension, pre-op beta blocker use, pre-op creatine, pre-op ejection fraction, history of smoking, and diabetes mellitus), cardiopulmonary bypass time, aortic cross-clamp time, case time, length of stay, transfusion requirements, complications (renal failure, prolonged ventilator sternal infection, and stroke), 30-day readmission, and 30-day mortality. Data on post-operative coronary angiography were obtained from the interventional cardiology database and chart review of the electronic medical record. This includes cardiac catheterization laboratory data from the main academic medical center and two community hospitals in our hospital system.

### 2.2. Definitions of Variables and Endpoints

Cardiopulmonary bypass time was defined as the total time on cardiopulmonary bypass during the case. Clamp time was defined as the total time the aortic cross-clamp was on during the case. Case time was defined as the time from first incision to skin closure. Blood transfusion was defined as receiving any transfusion of red blood cells, fresh frozen plasma, platelets, or cryoprecipitate during the case or post-operatively. Sternal infection was classified using the STS criteria of any superficial wound infection requiring antibiotics in the first 30 days. The STS risk score was calculated for each patient by the STS database using the criteria established by the STS [30]. Pre-operative ejection fraction was obtained from the pre-operative transthoracic echocardiogram. Prolonged ventilator support was defined as total support longer than 24 h. The only need for re-operation in the study was bleeding. Readmission was defined as readmission for any cause in the first 30-days post-op. Need for extracorporeal membrane oxygenation (ECMO) was defined as the need for ECMO at any point in the 30-day post-operative period. Need for an intra-aortic balloon pump (IABP) was defined as a pre- or post-operative intra-aortic balloon pump in the 30-day post-operative period. Angiographic graft stenosis on post-op catheterization greater than 50% was considered significant. 

### 2.3. Decision for TAR

The surgeon’s practice transitioned to TAR in August of 2017. TAR was the preferred procedure for all patients except for anatomical considerations including concerns about the arterial conduit such as size of the conduit or a failed Allen test.

### 2.4. Graft Harvesting Technique

The primary method for venous conduit harvest was an endoscopic technique. The radial artery was harvested with an open endoscopic technique, and the preferred technique for IMA harvest was skeletonized.

### 2.5. Exclusion Criteria

Exclusion criteria included off-pump, pump-assisted, single vessel, and emergent CABG. Patients that had CABG with a combination of multiple arterial grafts and any number of vein grafts were excluded from the study. Patients with only vein grafts were excluded. CABG using the gastroepiploic artery is not routinely performed at our institution and was not included in this study.

### 2.6. Institutional Review Board Approval and Informed Consent

This study was approved by the hospital Institutional Review Board on 14 June 2022 (Protocol #: 2022P0004540). The institutional review board granted an exemption from informed consent given the deidentified nature of the data and minimal risk of the study.

### 2.7. Statistics

Statistical analysis was performed using Prism 9 and Microsoft Excel 2021. Continuous data were analyzed using a *t*-test, or Mann–Whitney U test based on normality testing. Categorical data were analyzed using a chi-squared test or Fisher’s exact test when appropriate. Kaplan–Meier analysis using a log-rank test was performed on Prism 9 using the combined endpoint of any graft stenosis > 50% on heart catheterization or mortality. Operative time was plotted against case sequence to generate learning curves for TAR over time and analyzed using simple regression. The data were found to best follow a logarithmic curve. Data were broken down into two vessel, three vessel, and four vessel, as it is expected that the more anastomoses preformed the longer the case will be at baseline. 

## 3. Results

The query of the STS database for our institution identified 1075 patients who had CABGs performed by the operator from January 2014 through January 2022. After enforcement of the exclusion criteria, the study population included 898 total patients (458 IMA-SVGs and 440 TARs). After August 2017, the surgeon performed only 32 IMA-SVGs due to anatomical concerns. 

Baseline demographics were similar between groups. There was no significant difference in baseline age, sex, history of DM, history of hypertension, STS risk score, kidney function, or ejection fraction (all *p* > 0.05) (Table 1). The TAR group was less likely to have racial demographics reported and contained fewer smokers (*p* < 0.001, *p* = 0.03) (Table 1). The 30-day mortality was not significantly different with one mortality per group (*p* = 1.00). Although the TAR group had slightly longer clamp times (*p* = 0.002), cardiopulmonary bypass times (*p* < 0.001), and operative times (*p* < 0.001), ICU stay was shorter (*p* = 0.004), and 30-day readmission rate was lower for TAR compared with IMA-SVG (*p*= 0.001) (Table 1). The TAR group also required fewer postoperative transfusions (*p* < 0.001). There was no difference in renal failure, prolonged intubation, stroke, length of stay, or sternal wound complications between groups (all *p* > 0.05) (Table 1). At six months, one year, and two years there was a trend towards less graft stenosis in the TAR group (*p* = 0.17, *p* = 0.08, and *p* = 0.17) (Table 1).

The TAR group contained 440 cases. The preferred technique for IMA harvest was skeletonized with 433 (98.4) of 440 total cases (Supplementary Table S1). The TAR group included 322 (73.2) patients that received BIMA and 369 (83.9) patients that revied a radial artery (Supplementary Table S1). In total 243 (55.2) patients received both BIMA and a radial artery (Supplementary Table S1).

The rate of superficial sternal infection was 9 (2.8%) in the BIMA group and 2 (1.7%) in the non-BIMA group (*p* = 0.51). There was no difference in the rate of DM in the BIMA group with 187 (41.9%) patients in the BIMA and 61 (48.3%) patients in the non-BIMA group having DM (*p* = 0.23).

To evaluate smoking as a confounding factor on ICU times and the need for transfusion, we preformed subgroup analysis stratifying the data by smoking status. The stratification resulted in similar groups (Supplementary Table S2). Subgroup analysis showed that smoking did not impact the outcomes of ICU stay, transfusions, cardiopulmonary bypass time, clamp time, or case time (Supplementary Table S2). Among smokers there was a trend towards increased ICU stay in the IMA-SVG group (*p* = 0.05) and transfusions remained significantly elevated in the IMA-SVG group (*p* = 0.006) (Supplementary Table S2). In the non-smoking cohort, the ICU stay was significantly longer and transfusions more frequent in the IMA-SVG group compared to TAR (*p* = 0.02, *p* = 0.04) (Supplementary Table S2).

**Figure 1:**
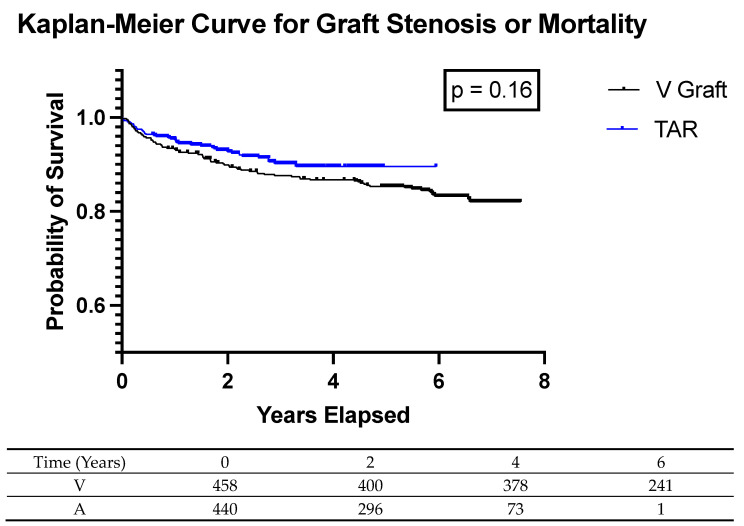
Kaplan–Meier Curve for Graft Stenosis or Mortality. Kaplan–Meier analysis using the combined endpoint of graft stenosis on heart catheterization or mortality shows a trend towards decreased events in the total arterial revascularization (TAR) group compared to one internal mammary artery and vein grafts (V Graft). The accompanying at risk table shows the at-risk patients at each time point. A, total arterial grafting group; V, vein graft group.

**Figure 2:**
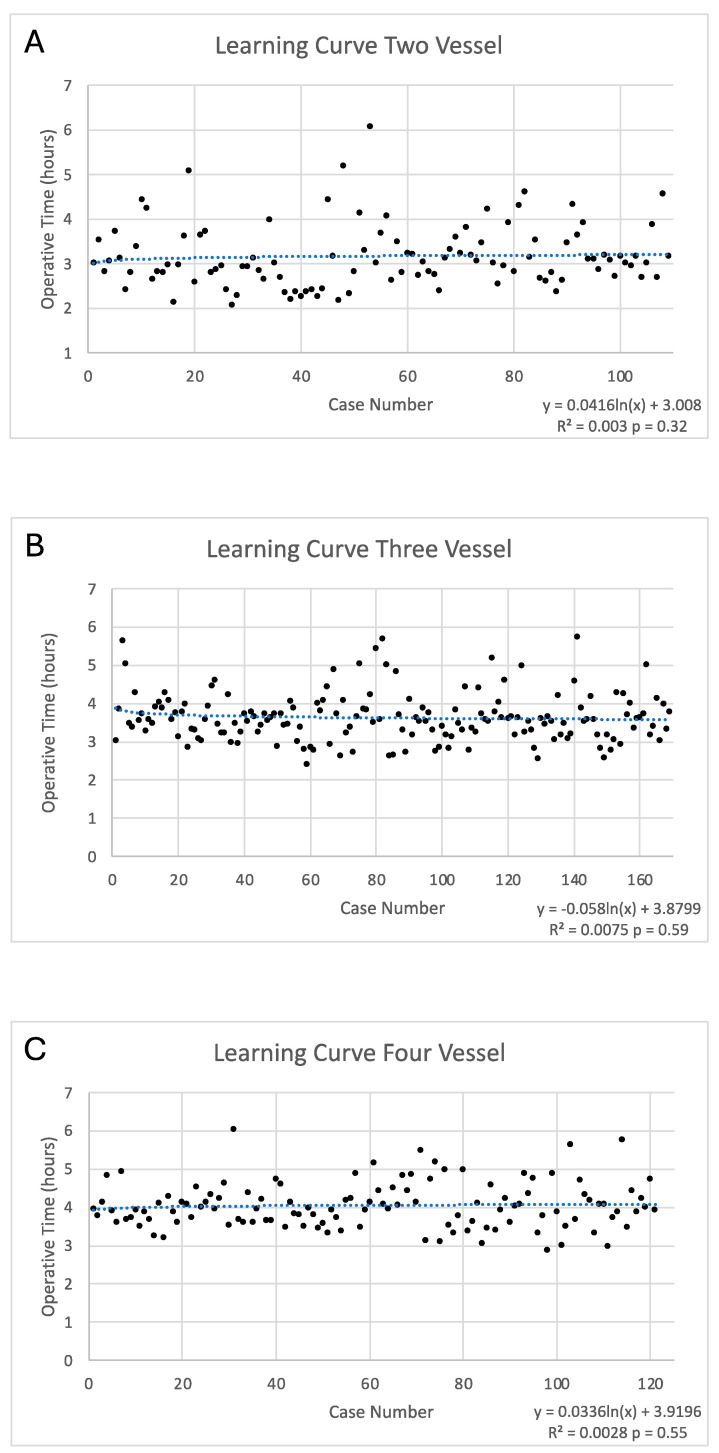
Learning Curve for Total Arterial Revascularization. (**A**) shows the learning curve for two vessel total arterial revascularization (TAR). There was no significant learning curve with two vessel TAR using linear regression. (**B**) shows the learning curve for three vessel TAR. There was no significant learning curve with three vessel TAR using linear regression. (**C**) shows the learning curve for four vessel TAR. There was no significant learning curve with four vessel TAR using linear regression.

Kaplan–Meier curves analyzing any one graft with > 50 graft stenosis and mortality showed a trend towards a decrease in the combined endpoint in TAR (*p* = 0.16) (Figure 1). The average TAR was 30 min longer than traditional IMA-SVG; however, learning curves, stratified by number of bypasses, showed no significant learning curve associated with TAR (Figure 2). The association of two vessel CABG with case order in time had an R squared value of 0.002 and a *p* value of 0.32 (Figure 2). The association of three vessel CABG with case order in time had an R squared value of 0.0075 and a *p* value of 0.59 (Figure 2). The association of four vessel CABG with case order in time had an R squared value of 0.0028 and a *p* value of 0.55 (Figure 2).

## 4. Discussion

Despite multiple clinical studies demonstrating long-term benefits of TAR, TAR remains underutilized in clinical practice [19,20,21,22]. The underutilization of TAR is due to concerns such as sternal wound infections, patient selection, and the learning curve. In an effort to better understand the learning curve associated with TAR, we present the largest single-surgeon experience focused on learning curve and short-term outcomes. The results of our study show that TAR has similar short-term outcomes to IMA-SVG with a minimal learning curve.

The patients undergoing TAR had increased cardiopulmonary bypass, aortic cross clamp times, and case times. This increased time is likely related to the technical difficulty associated with arterial grafting. The increase in pump and cross clamp times is likely related to the increased complexity of preforming anastomoses with smaller arterial grafts compared to venous grafts. The increased total case time is likely related to an increase in pump and cross clamp times as well as the increased complexity of harvesting arterial grafts [31]. There appeared to be no significant learning curve associated with TAR as the case time did not change significantly with increasing experience for two, three, or four vessel CABG. Importantly, this increase in case time does not appear to have an impact on complications.

We found no difference in several short-term outcomes including renal failure, prolonged intubation, stroke, length of stay, sternal wound complications, or mortality between the TAR and IMA-SVG groups. The lack of a difference in sternal wound complications is important as one of the major concerns regarding the use of TAR is the potential for increase in sternal wound infections due to devascularization of the chest wall with use of the LIMA and right internal mammary artery. This is further validated by our small subgroup analysis comparing BIMA and non-BIMA patients showing no increased risk of sternal would infection with BIMA. It has been reported in several studies that TAR increased the risk of sternal wound infections; however, our data suggest that a single center can perform TAR without a significant increase in risk [32,33]. It is also important to note the TAR group had a decrease in time in the ICU and a decrease in the need for blood transfusions, independent of smoking status. This would imply that an experienced surgeon transitioning to total arterial grafting has no increase in risk for major complications and may benefit patients by decreasing ICU time and transfusion associated complications [34,35,36]. It is particularly important when looking at transfusion requirements and ICU times to consider shifting practice over the last 10–15 years in cardiac surgery. Literature and society guidelines have pushed many surgeons to avoid transfusions and to limit total time in the hospital, and this could explain the decrease in transfusions and ICU times seen in our study. Given that only 32 IMA-SVG occurred after transitioning to TAR, it is impossible to fully investigate the effects of time on transfusion requirements and ICU times.

TAR has been associated with decreased cardiac events and increased graft survival in prior studies [37,38]. In our study, we found a trend towards decreased graft stenosis at six months, one year, and two years. We also had a trend towards a decrease in the composite endpoint of graft stenosis and mortality using Kaplan–Meier analysis. This is encouraging for the TAR group; however, it is important to remember that these data are limited by follow-up and a time delay. Many benefits of TAR may not be seen for 5–10 years and this is far beyond the follow-up of this study.

The results of our study suggest that an experienced surgeon transitioning to TAR can expect increased case time, but the learning curve is small. More importantly, this increase in case time is not associated in with an increase in major complications. TAR showed a strong trend towards decreased graft stenosis and long-term mortality. This single surgeon experience further validates the growing body of literature that TAR is a safe, and potentially more effective, form of CABG for eligible patients. Further work is needed to analyze the learning curve associated with TAR, but we believe that our results combined with the current body of literature should encourage experienced surgeons to transition to TAR as their preferred technique for CABG.

This study has several important limitations. The study compares overall well-matched groups; however, this is a baseline difference in racial demographics and smoking status. The baseline racial demographics are skewed by an increase in non-reporting in the TAR group. This started after 2020 and is likely related to staff turnover and the COVID-19 pandemic. This limits the generalizability of the study and makes it difficult to truly know how similar racial demographics are between groups. Subgroup analysis suggest that smoking status does not impact outcomes. The retrospective nature and lack of randomization make it more prone to potential bias or confounding factors. The TAR cases were performed more recently than most of the IMA-SVG cases. The surgeon studied has not had drastic changes in his post-operative management during the study time, but it is impossible to exclude possible confounding factors from changes in peri-operative care independent from the surgeon, such as changes in nursing, anesthesiology, or intensive care unit staff during this time. This is particularly important when discussing outcomes such as ICU time and transfusion that might have been impacted by subconscious changes in practice. This study is limited to IMA-SVG and TAG and results might be different in the case of multiple cardiac operations.

## 5. Conclusions

An experienced surgeon transitioning from IMA-SVG to TAR slightly increases operative time, but decreases ICU stay, readmissions, and postoperative transfusions with no significant difference in rates of immediate post-operative complications including sternal wound infections or 30-day mortality. The learning curve for an experienced surgeon transitioning to TAR is minimal. Furthermore, short-term results show a trend towards increased graft patency with TAR.

## Figures and Tables

**Table 1:** Coronary Artery Bypass Grafting Surgeries Demographics and Outcomes. A, total arterial grafting group; DM, diabetes mellitus; ECMO, extracorporeal membrane oxygenation; IABP, pre- or post-operative intra-aortic balloon pump; Re-op, any reoperations in first 14 days; STS, Society of Thoracic Surgeons; V, vein graft group; % for Cath with Stenosis is *n* over the number of patients that have made it to that post-operative time point.

	A (440)	V (458)	*p*
Age (years, mean (SD))	66.6 (9.7)	66.9 (9.9)	0.70
Sex Male, *n* (%)	360 (81.8)	363 (79.3)	0.94
Racial Demographics			
White Non-Hispanic, *n* (%)	281 (63.9)	369 (80.6)	<0.001
White Hispanic, *n* (%)	14 (3.2)	20 (4.4)	
African American, *n* (%)	33 (7.5)	28 (6.1)	
Asian, *n* (%)	10 (2.3)	16 (3.5)	
Native American, *n* (%)	3 (0.7)	7 (1.5)	
Pacific Islander, *n* (%)	1 (0.2)	1 (0.2)	
Other/Unknown, *n* (%)	98 (22.3)	17 (3.7)	
STS Risk Score (Median [IQR])	0.009 [0.005–0.017]	0.010 [0.005–0.021]	0.17
History of Smoking, *n* (%)	230 (52.3)	277 (60.5)	0.01
History of DM, *n* (%)	192 (43.6)	203 (44.3)	0.84
History of HTN, *n* (%)	369 (83.9)	383 (83.6)	0.92
Pre-op Beta-blocker	398 (90.5)	421 (83.6)	0.44
Pre-op Creatinine	1.0 [0.8–1.1]	1.0 [0.8–1.2]	0.94
Pre-op Ejection Fraction	52.5 (11.6)	52.2 (12.2)	0.73
Urgent Case, *n* (%)	282 (64.1)	282 (61.6)	0.43
30-day Mortality, *n* (%)	1 (0.2)	1 (0.2)	1.00
CABG Vessel Number			
Distal Anastomosis (Median [IQR])	3 [3–4]	3 [3–4]	0.16
Two Vessel, *n* (%)	109 (24.8)	105 (22.9)	0.64
Three Vessel, *n* (%)	169 (38.4)	163 (35.6)	
Four Vessel, *n* (%)	121 (27.5)	136 (29.7)	
Five Vessel, *n* (%)	36 (8.2)	48 (10.5)	
Six Vessel, *n* (%)	5 (1.1)	6 (1.3)	
Cardiopulmonary bypass time (minutes, Median [IQR])	84 [67–101]	76 [64–91]	<0.001
Clamp Time (minutes, Median [IQR])	72 [57–87]	65 [53–77]	0.002
Case Time (hours, Median [IQR])	3.65 [3.18–4.13]	3.17 [2.75–3.68]	<0.001
Time in ICU (hours, Median [IQR])	33.0 [25.5–56.5]	46.2 [26.2–74.0]	0.004
Renal Failure, *n* (%)	5 (1.1)	5 (1.1)	1.00
Prolonged Ventilator, *n* (%)	21 (4.8)	28 (6.1)	0.54
Sternal Infection, *n* (%)	11 (2.5)	9 (2.0)	0.65
Stroke, *n* (%)	5 (1.1)	4 (0.9)	0.75
30-day Readmission, *n* (%)	39 (8.9)	73 (15.9)	0.001
Re-op, *n* (%)	11 (2.5)	14 (3.1)	0.61
IABP, *n* (%)	24 (5.5)	28 (6.1)	0.67
Need for ECMO, *n* (%)	0 (0.0)	1 (0.2)	1.00
Post-op Stay (Days, Median [IQR])	5 [4–7]	5 [4–6]	0.05
Need for Transfusion, *n* (%)	111 (25.3)	165 (36.0)	<0.001
Angiographic Graft Stenosis > 50% at 6 Months, *n* (%)	8/440 (1.8)	15/458 (3.3)	0.17
Angiographic Graft Stenosis > 50% at 1 Year, *n* (%)	11/407 (2.7)	23/456 (5.0)	0.08
Angiographic Graft Stenosis > 50% at 2 Years, *n* (%)	17/327 (5.2)	36/444 (8.1)	0.17

## Data Availability

Data are available on reasonable request to the corresponding author.

## Supplementary Materials

The following supporting information can be downloaded at: https://www.mdpi.com/article/10.3390/jcm13164831/s1.
