# Supplementary material for: A Single-Surgeon Experience Transitioning to Total Arterial Revascularization
Source: J Clin Med. 2024 Aug 16;13(16):4831. doi: 10.3390/jcm13164831 (PMC11355799; doi:10.3390/jcm13164831)
Supplement: Supplementary file 1 [file jcm-13-04831-s001.zip › jcm-3163455-supplementary.pdf]

Journal of
Clinical Medicine

MDPI

Supplemental Table S1. Total Arterial Revascularization Group Data. A, total arterial grafting group; BIMA bilateral internal mammary artery.

	A (440)
BIMA	322 (73.2)
Radial Graft	369 (83.9)
BIMA + Radial	243 (55.2)
Skeletonized	433 (98.4)

Supplemental Table S2. Smoking Status Subgroup Analysis. A, total arterial grafting group; DM, diabetes mellitus; STS, Society of Thoracic Surgeons; V, vein graft group.

	Smoking			Non-Smoking		
	A (230)	V (277)	p	A (210)	V (181)	p
Age (years, mean (SD))	67.8(9.3)	68.1(9.1)	0.70	65.4 (10.1)	65.0 (10.7)	0.74
Sex Male, n (%)	189 (82.2)	222 (80.1)	0.56	171 (81.4)	141 (77.9)	0.39
Risk Score (Median [IQR])	0.017 [0.006-0.02]	0.020 [0.0062-0.023]	0.67	0.008 [0.005-0.016]	0.008 [0.005-0.016]	0.36
History of DM, n (%)	102 (44.3)	120 (43.3)	0.85	90 (42.9)	83 (45.9)	0.55
Distal Anastomosis (Median [IQR])	3 [3-4]	3 [3-4]	0.18	3 [2-4]	3 [2.5-4]	0.67
Cardiopulmonary bypass time (minutes,Median [IQR])	84 [67-98]	78 [65-92.5]	**0.001**	84.5 [67-103]	73 [61.5-87]	**< 0.001**
Clamp time (minutes, Median [IQR])	72 [98-67]	66 [53-80]	**0.007**	72 [56-88.25]	63 [23]	**< 0.001**
Case Time (hours, Median [IQR])	3.6 [3.2-4.1]	3.2 [2.8-3.7]	**<0.001**	3.7 [3.2-4.2]	3.2 [2.6-3.6]	**< 0.001**
Time in ICU (hours, Median [IQR])	32.7 [25.2-60.4]	45.1 [71.9-26.1]	**0.05**	32.7 [25.8-56.9]	47.5 [26.2-76.4]	**0.02**
Need for Transfusion, n (%)	59 (37.1)	103 (37.2)	**0.006**	52 (24.8)	62 (34.3)	**0.04**
